# Reliable Narrators of Experience: Rethinking Dementia Narratives from Insider Perspectives

**DOI:** 10.1002/hast.4989

**Published:** 2025-09-18

**Authors:** Kate de Medeiros

**Keywords:** chaos narratives, tripartite self, illness narratives, dementia, small stories, broken stories, bioethics

## Abstract

Cultural narratives about dementia reinforce the idea that people living with the condition are unreliable narrators of their own experiences. Challenges with recall and memory and changes in language that are commonly experienced by people living with dementia become equated to a loss of self. Since language is a shared space where people construct meaning through stories, stories that lack coherence or exhibit broken language are often discounted. To counter the notion that broken narratives reveal broken selves, I present a story by Marlene, a person living with dementia who recalled an encounter with a bobcat while on a camping trip. Rather than considering the veracity of her story, I focus on the importance of the emotions presented—the feeling self. Overall, I argue that by shifting focus from story challenges to expressed emotions, we are better positioned to understand and respect people living with dementia as authorities of their own experiences.

Cultural narratives about dementia reinforce the idea that people living with the condition are unreliable narrators of their own experiences and at risk of losing selfhood.^1^ Contributing to dementia cultural narratives are assumptions about personal narratives. Personal narratives have long been assumed to be a key component of self and identity; ordering the autobiographical events in one's life to reflect, relay, consider, and respond is typically viewed as important if not essential to personal identity. Difficulty with word and memory recall and decreasing cohesion in discourse—hallmark symptoms of Alzheimer disease and other dementias—become equated with “loss of self.” In addition, mistakes in autobiographical details or language (such as grammar and syntax) by people living with dementia result in their stories being discounted and delegated to others (such as friends, spouses, and other family members) to tell. These and other assumptions raise two questions: Can an outsider really understand and narrate the experience of living with dementia? And how can we rethink narrative practices to recognize the many ways that selves are expressed beyond those ways that are rooted in autobiographical memory?^2^

In this essay, I consider how cultural narratives about dementia influence who gets to narrate the experience of living with dementia. I begin by considering the role of memory and recall in narratives. Next, I discuss what cultural and personal narratives reveal about values and how they inform each other. Finally, I consider how the emotional memory expressed in a small story by someone I call Marlene, a person living with dementia, resists many dominant cultural narratives of dementia and points to ways to better understand expressions of selfhood and the experience of living with dementia. While it is important to note that narratives are not limited to spoken language, my focus here will be on language‐based narratives.

## Dementia Stories and Their Narrators

Cultural narratives are stories embedded within a culture that reinforce norms, values, expectations, roles, and interpretations of meaning through unspoken and deeply engrained rules. A couple of dominant cultural narratives about dementia, for example, characterize dementia as a “long goodbye” or convey that people with dementia are “strangers” to themselves and loved ones. Cultural narratives provide a source of content, genres, and plotlines that heavily influence which stories are told and which are silenced, which stories are valued and which are discounted, and who gets to tell a particular type of story and why.^3^

Consider, for example, dementia stories in the genre of illness narratives. With some notable exceptions, dementia stories are typically told from outsiders’ perspectives rather than by people living with dementia (see Wendy Hulko's work for an excellent consideration of dementia narratives^4^). Examples include memoirs such as *All Gone: A Memoir of My Mother's Dementia. With Refreshments* (2012), by Alex Witchel, and *Where the Light Gets In: Losing My Mother Only to Find Her Again* (2017), by Kimberly Williams‐Paisley, and novels such as *Still Alice* (2009) and *Turn of the Mind* (2011), by Alice La Plante, and *The Swimmers* (2022), by Julie Otsuka. Common features of outsider dementia stories include a clear linear progression of action, attention to accuracy of details, and depth of introspection. As many writers of outsider dementia narratives explain, telling stories about their experiences with dementia gives them and their readers a way to better understand and empathize with people living with dementia.

While these and other works provide important insights into the real or imagined experiences of others, they also may reinforce dementia cultural narratives about fading selves and looming nothingness, as words like “gone” and “losing” in a couple of the titles suggest. When writers speak on behalf of people living with dementia, the experience of living with dementia is ventriloquized, often because, as Heike Hartung and colleagues have observed, it is assumed that “since dementia patients can no longer speak for and of themselves—at least not in the ways ordained by our culture—others must take over the job.”^5^ In such cases, the overall message is that others must repair the broken narratives of people living with dementia due to challenges associated with language and cohesion.

Of course, there are notable narratives by people living with dementia—particularly early‐onset forms of dementia—as well. For example, Richard Taylor's book *From the Inside Out* (2006), a collection of essays about his experience as someone living with early‐onset Alzheimer disease, provides a first‐hand account of his observations and experiences. Taylor writes, “From my perspective as a person living with the diagnosis, there is far too much emphasis on the label, the name, and the symptoms associated with the disease and too little emphasis on the individuals who actually *have* the disease.”^6^ Daniel Gibb and Teresa Barker offer another firsthand perspective: *A Tattoo on My Brain: A Neurologist's Personal Battle against Alzheimer's Disease* (2021) provides a neurologist's point of view of his experience with early‐onset Alzheimer disease. *Somebody I Used to Know* (2019), by Wendy Mitchell, written with Anna Wharton, is a personal account of Mitchell's diagnosis of young‐onset vascular dementia Alzheimer disease at age fifty‐eight and subsequent experiences. There are two voices present throughout. The “I” describes her experiences in the present (as with “I glance around the table at the faces I know are familiar, and yet I can't recall their names”).^7^ The “you” is for reflective comments she makes to herself (such as, “You weren't the kind of person who forgot anything.”)^8^

While a simple internet search reveals pockets of stories here and there from the perspective of people living with dementia, these stories are difficult to find. In addition, when stories are available, as with the previous examples, they are typically extremely well written and polished, giving them important credibility. They also tend to come from people who enjoy social advantages based on their personal identities. An observation by Hulko more than twenty years ago holds true today: “We have not heard from traditionally marginalized groups of people such as ethnic and ‘racial’ minorities, lesbians/gays/bisexuals, poor people, and uneducated people.”^9^ Such omissions in the genre of personal dementia narratives suggest the need for counternarratives that capture a wide variety of experiences and forms of telling that go beyond the polished story and include “broken stories,” which I discuss later.

## Selfhood and Dementia

Before considering personal narratives in more detail, I consider selfhood in the context of dementia and cultural narratives. As mentioned, cultural narratives about dementia equate challenges with recall and memory and changes in language use to a loss of self. However, what constitutes loss of self is less clear. In a systematic review of self in dementia, Lisa Caddell and Linda Clare found that self was often not explicitly defined but rather implicitly understood, depending on the epistemological paradigm of the author(s).^10^ For example, some studies considered self to be reliant on a clear narrative act or on one's ability to tell a cohesive life narrative. Other studies considered any telling of a life narrative, regardless of size or complexity, to demonstrate preservation of self. The latter idea is illustrated in Steven Sabat and colleagues’ social constructionist model of the “tripartite self.”^11^ In this model, selves are constructed and enacted through language and social encounters. For Sabat and coauthor Michelle Collins, Self 1 encompasses personal identity and the daily experience of being a unique and individual person, often expressed through an indexical “I.”^12^ Self 2 comprises physical characteristics (such as eye color) and mental attributes such as accomplishments, beliefs, attitudes, and internal evaluations (for example, Self 1 claims, “I am an accomplished person,” whereas Self 2 expresses subjective emotions about personal accomplishments, as with “I feel proud to be an accomplished person”). Self 3 is one's public persona (their roles, for example) maintained through social interactions with others (illustrated by thoughts such as “I am seen by others as a leader in my field.”) Caddell and Clare found that, in many studies using this tripartite self‐schema, there is support that all three selves remain present throughout the course of dementia, an important consideration with regards to narratives.^13^ People expressing a narrating “I” could reflect on their beliefs, attitudes, and feelings and could counter or respond to outsiders’ valuations of their current selves living with dementia. Yet, despite finding support for the presence of self, the authors also report that autobiographical memory loss could contribute negatively to Self 2 and Self 3. For example, a person may talk about feeling shame or embarrassment when experiencing difficulties with recall (part of Self 2) or of being treated as incapable when given the label of having dementia (part of Self 3).

Other conceptions of self that were identified by Caddell and Clare include interactionist perspectives, in which selves are understood to be constructed, communicated, and maintained through interactions with others;^14^ embodied selfhood, which concerns how selfhood resides in the body and is communicated through gestures and other actions;^15^ and the narrated self, mentioned earlier, which considers how selves are communicated through personal narratives. While the description of personal narratives was limited in Caddell and Clare's review, it is a topic of special interest here.

## Personal Narratives

Personal narrative” is an umbrella term describing a group of discursive acts (big and small stories) that reveal some aspects of the culturally constituted individual.^16^ Examining narrative features, such as what details are included and omitted, can help one uncover and challenge larger cultural narratives at work. For example, why this particular story now? Why these details and not others? Why these words and metaphors? Who is present, and who is missing from the cast of characters?

As Alison Phinney and others have observed, there has been a long‐standing assumption that people bring order and understanding to their worlds through storying their experiences, stories are a necessary way to reflect on one's life, and the self is contingent on narrative identity.^17^ Since language is a shared space where people construct meaning with others through stories, broken language (unconventional syntax, inappropriate word use, and grammatical incoherence, for example) has been mistaken for a sign of a broken self. Because people with dementia are often not recognized as having a self to express, their stories may be ignored or discarded.

There are two broad categories of personal narratives: big stories and small stories. Big stories are the well‐formed, often rehearsed, overarching storylines to which people align their biographical experiences. They include the grand life narratives that psychologist Dan McAdams describes as aligning to archetypes.^18^ For example, “the redemptive self” storyline communicates a Benjamin Franklin‐like resolve toward personal success despite adversity, and the “I was chosen” story aligns personal accomplishments with some special gift or unique advantage. Other examples of big stories include the outsider dementia memoirs mentioned earlier that borrow forms (such as first‐person memoir) and plots (dementia has taken away my parent, for example) from existing cultural stores of possibilities, which, in turn, shape their content.

In contrast, small stories are often unrehearsed and presented as an aside or divergence from a bigger story.^19^ They may exist as incomplete ideas or partial observations and may bear similarities to what Lars‐Christer Hydén calls “broken stories” when referring to stories by people living with dementia.^20^ Insider dementia stories, according to Hydén, “are perceived to be broken and fail because in contrast to other stories they are fragmented, partial, jumbled, and repetitive; they lack temporal and thematic coherence, and shift between characters, places, and times without any notice.”^21^ They also may lack outside confirmation of accuracy.

In addition, since emotional experiences and memories of people living with dementia may not lend themselves to well‐polished, linear narratives, their stories may fall into what Arthur Frank calls “chaos narratives.”^22^ A chaos narrative is a type of antinarrative; due to profound physical, mental, or emotional pain, the person is not able to gain a reflective distance from their experience to create either a restitution narrative (in which, the person is restored to health, for instance) or a quest narrative (in which, in Frank's formulation, the person reflects on their experiences and shares the knowledge they have gained with others, as in the dementia memoirs mentioned earlier) narrative. Chaos narratives are therefore often discarded. Yet, as Colleen Donnelly has argued in reference to disability narratives in general, not dementia narratives specifically, “The consumer literary market still remains largely unreceptive to writers who choose not to shape their narratives to accepted ableist conventions and tastes that audiences have been trained to cultivate.”^23^

Consider the following small story by a person living with dementia in a long‐term care facility who participated in a poetry program. Marlene told this story in response to the facilitator's question whether anyone had ever been camping: “I like camping. I like waking up in the middle of the night when nobody else in the camp is awake. And just listening. And you hear sometimes, like wild cats. Something like that. And one time I heard a crack. And I look up; my eyes looked. And there was a bobcat. Just sitting there. So I sat up real slowly and nodded at him. He nodded. I put my hand out, and he licked it. My husband said, ‘Where's the bite marks?’ I said, ‘I didn't get bitten.’ [laughs]. He says, ‘Just keep your hands in your pockets.’ I said, ‘My pajamas don't have pockets.’ But he [the bobcat] was as interested in me as I was in him.” Marlene's story is well formed in many respects—there is a temporal ordering of events, a plot, a complicating action, an evaluation. However, some readers may question details in the story, especially after learning that Marlene is living with dementia: Could such a story be true? Has Marlene confused some of the details? Was her husband present on the camping trip when this encounter happened? How could she escape unhurt with a bobcat so close? Is there anyone who can corroborate the details? And so on. Consequently, Marlene's story would likely be considered broken as a result of “confusion” or “confabulation,” which also serve as evidence of her fading self. Her story may also be subject to higher levels of scrutiny than the same story told by someone who did not have a diagnosis of dementia.

Here, I pause to consider what it means to be a reliable narrator of experience. I am not drawing from the extensive work in literary studies regarding reliable and unreliable narrators in fiction since fictional characters differ from real people in social contexts. Instead, I consider some of the social science work that takes up the idea of narrative identity in the context of living with dementia. Marie Mills posed the challenge as follows: “A narrative identity, which is supported by personal knowledge of one's individual biography or life history, presupposes two fundamental conditions. Firstly, that one has accrued a life history and, secondly, that it is remembered. It is this latter condition which suggests that the study of narrative identity in dementia may be problematic, based as it is on the recall of the life story.”^24^ Mills also helpfully points out that such a limited view on narrative identity focuses solely on the cognitive self rather than on the “feeling” self, something that the previous conceptions of self also did not directly address. As Mills and others have argued, by ignoring the importance of emotional memories, we reduce the person living with dementia to the sum of the autobiographical facts that they can recall instead of seeing them as authorities of their emotional experiences.^25^

Just like with any story, we can read and interpret Marlene's story in many ways. At face value, it is a story about an encounter with a bobcat on a camping trip. However, reading Marlene's story in terms of emotional events rather than as a series of verifiable autobiographical memories presents new possibilities for understanding. By considering questions about the content of narratives posed earlier in the essay (Why this particular story now? Why these details and not others? Why these words and metaphors? Who is present, and who is missing from the cast of characters?), we can interpret Marlene's story in light of the series of events she tells.

For example, the moment Marlene describes is a quiet time of night, “when no one else is awake.” Marlene is alone and mentions how much she loves the quiet. Dementia‐care facilities are noisy places, where there is always some type of activity or noise. Perhaps Marlene experienced a pause in the very chaotic world of dementia care. In the pause, there was a looming danger that Marlene could see—a bobcat (or dementia? the care staff? her future?). Marlene confronts her fear by nodding and then reaching out to the bobcat, who, in turn, nods and licks her hand. The image she conveys is comforting. She describes the bobcat as “just as interested in me as I was in him.” Her husband, who is a character in her story but does not witness the event, does not believe her and wants to see the bite marks, telling her, “Keep your hands in your pockets.” Yet, as Marlene points out, her pajamas do not have pockets, or there are no places for her to hide or, as it turns out, any need to be afraid. In many ways, Marlene's story could be interpreted as one of strength and agency despite her living in a frightening place—the nursing home—with a condition that makes others see a person as unreliable.

## The Feeling Self and Firsthand Experience

Here, I have considered the ways that personal narratives of people living with dementia can be silenced by cultural narratives that position them as unreliable narrators of their own experiences. Although, over the course of the illness, it may become increasingly difficult for people living with dementia to recall specific autobiographical memories (of events, people, places, and so forth), they are still able to use stories to express emotional memories or the *feeling self*, and their stories remain important and worth attending to. Many outsider dementia stories try to capture the emotional aspects of dementia to build empathy, yet the insider stories by people best able to communicate this experience (people living with dementia) are left out. The future focus of narrative approaches to understanding dementia should be on narratives of the feeling self.^26^ It would have been helpful, for example, to have asked Marlene to talk more about her experience with the bobcat: “What did it feel like to recall that story?” “What would you do if you saw the bobcat now?” “Is there anything you'd like to add to the story?” These and other questions could be an important way to move past the “facts” and dig deeper into the emotions.

One could also ask Marlene directly about her experience living in the nursing home: “What fears do you have?” “What strengths do you experience?” “Do you feel loved?” Often, questions that involve emotions are not asked, perhaps because the potential listeners fear that such questions would upset either the tellers or themselves. To counter dominant cultural narratives about the experience of living with dementia, we need to be open to narratives created by people living with dementia, with openness both to interpretation and to respecting the narrators as the authorities on their own experiences.
